# Long‐Term Follow‐Up of Patients With Transaldolase Deficiency

**DOI:** 10.1002/jimd.70173

**Published:** 2026-03-13

**Authors:** M. Scaglione, A. Brassier, A. Wiedemann, I. Jankowska, J. Pawłowska, T. Lamireau, L. Bridoux‐Henno, C. Douillard, T. Casswall, U. Cucinotta, M. Gaschignard, P. Socha, F. Feillet, B. Fischler, D. Samara‐Boustani, M. C. Schiaffino, M. Schiff, P. De Lonlay, F. Lacaille

**Affiliations:** ^1^ Reference Center for Inborn Errors of Metabolism, Department of Pediatrics Necker‐Enfants‐Malades Hospital, APHP, University of Paris Cité, French G2m Network for Metabolic Diseases, MetabERN, INSERM‐1151, INEM, INSERM UMR _S1163, Institut Imagine Paris France; ^2^ Department of Neuroscience, Rehabilitation, Ophthalmology, Genetics, Maternal and Child Health University of Genoa Genoa Italy; ^3^ Department of Paediatrics Hôpital d'Enfants Brabois, CHU Nancy Vandoeuvre les Nancy France; ^4^ Department of Gastroenterology, Hepatology, Nutritional Disorders and Pediatrics The Children's Memorial Health Institute Warsaw Poland; ^5^ University Hospital of Bordeaux Bordeaux France; ^6^ Department of Child and Adolescence Medicine Sud Hospital Rennes France; ^7^ Department of Endocrinology, Diabetology and Metabolism Reference Center for Inherited Metabolic Disease, Metab ERN, CHU Lille Lille France; ^8^ Department of Pediatrics, Astrid Lindgren Children's Hospital Karolinska University Hospital, CLINTEC, Karolinska Institutet Stockholm Sweden; ^9^ Pediatric Hepatology and Gastroenterology‐Nutrition Necker‐Enfants Malades University Hospital Paris France; ^10^ Department of Pediatric Endocrinology Necker‐Enfants Malades Hospital Paris France; ^11^ Department of Pediatrics IRCCS G. Gaslini Genoa Italy

**Keywords:** kidney transplantation, liver failure, liver transplantation, multiorgan involvement, pentose phosphate pathway, transaldolase deficiency

## Abstract

Transaldolase (TALDO) deficiency has a well‐characterized phenotype. However, there are few large cohort studies, and little is known about the long term, including the need for organ transplantation. Our aim was to share a long multicenter experience in managing these patients. We retrospectively collected data on 16 patients followed for up to 20 years. Liver involvement was present in all patients, with six presenting liver failure at birth. During long‐term follow‐up, 10 patients developed cirrhosis and four underwent liver transplantation (LTx). Two patients died from severe liver failure in the first months of life; persistent weight below 4 kg precluded LTx. A third patient died from posttransplant complications at 6 months of life. Five patients developed chronic kidney failure, and in two of them kidney transplantation was considered after the age of 20 years. Patent foramen ovale (PFO) was the most frequent cardiac finding; cardiomyopathy or ventricular dysfunction was diagnosed in five patients. Twelve patients developed chronic cytopenia, all presenting with thrombocytopenia. Hypergonadotropic hypogonadism was found in seven patients; two others developed secondary adrenal cortisol insufficiency and primary hypothyroidism requiring replacement therapy. Mild neurocognitive delay was observed only in a few cases. We described different patterns of liver disease progression. Transplantation should be early considered if indicated for liver insufficiency; in the most severe patients with neonatal onset, achieving sufficient weight for transplantation may be challenging. Our preliminary data suggest that, in older patients, kidney involvement may also warrant consideration of kidney transplantation, which was not previously described for this disease.

## 1 Introduction

Transaldolase deficiency is a rare autosomal recessive metabolic disorder (OMIM #606003) caused by pathogenic variants in the *TALDO* gene. This gene encodes the enzyme transaldolase, which catalyzes reversible reactions in the pentose phosphate pathway (PPP), a key metabolic route in which glucose‐6‐phosphate (G6P) is converted to ribose‐5‐phosphate (R5P). The irreversible part of this pathway is rate‐limited by the activity of glucose‐6‐phosphate dehydrogenase (G6PD).

To date, fewer than 50 cases of transaldolase deficiency have been reported in the literature. Clinical manifestations are primarily hepatic, with hepatosplenomegaly as a hallmark, but may also include pancytopenia, bleeding tendencies, growth retardation, dysmorphic features, cutis laxa, congenital heart disease, kidney disease, and hypogonadism [1, 2]. Liver involvement is present in all reported patients and ranges from isolated hypertransaminasemia to acute liver failure, cirrhosis, and hepatocellular carcinoma (HCC).

TALDO deficiency is probably underdiagnosed, as it can cause in utero death, neonatal death, or cryptogenic cirrhosis. Most current knowledge derives from case reports or small cohorts, with limited information on long‐term outcomes and management.

There is no etiological treatment; organ‐specific complications are managed individually, and the indications and timing for organ transplantation remain uncertain. Our aim is to describe the long‐term natural history and organ involvement in TALDO deficiency, providing insights into the potential role of liver and kidney transplantation (Tx).

## 2 Patients and Methods

In this retrospective study, we included patients with a confirmed diagnosis of TALDO deficiency who were followed at various European centers between 1999 and 2025: Necker–Enfants malades Hospital in Paris, Sud Hospital in Rennes, Huriez Hospital in Lille, Bordeaux Hospital, Karolinska University Hospital in Stockholm, and the Children's Memorial Health Institute in Warsaw.

For each patient, clinical data were collected during periodic evaluations, appropriate for their age, using both written and electronic medical records.

Recorded data were family history, pregnancy, birth symptoms, laboratory tests (AST, ALT, γ‐GT, total bilirubin, prothrombin time [PT], albumin, complete blood count, and creatinine), genetic analyses, and liver, kidney, hormonal, cardiac, neurological, and other organ involvements.

## 3 Results

We collected data on 16 patients (12 boys), 12 of whom were from consanguineous families, including five sibling pairs (Table 1). At last follow‐up (FU), 13 were alive.

**TABLE 1:** Patients' characteristics.

All/living patients at last FU	*N* = 16/13
	**Median**
Age at last FU (years;13 patients)	17.6 (IQR 14.8–20.3)
Age at death (months; 3 patients)	3.0 (IQR 2.75–4.5)
Age at diagnosis	8 months (1 month–22 years)

First signs of disease	*N* (%)
Intrauterine growth restriction	13 (81)
Facial dysmorphism	12 (75)
Cutis laxa/wrinkled skin	13 (81)
Telangiectasia	6 (38)
Hypertrichosis	5 (31)

Four additional patients with TALDO deficiency were excluded because of limited data: one fetus with hydrops and in utero death, and a 4‐month‐old infant, siblings of Patients 13 and 14; and two siblings from Saudi Arabia who both died in the first months of life.

Genetic data were available for 11 patients; in Table 2 the association between genetic variants and long‐term outcome is reported.

**TABLE 2:** Genetic data and long‐term outcome.

Patient (No.)	TALDO gene variant	Sex (M/F)	Age at clinical onset (months)	Long‐term outcome
1	c.575G>A (hom.)	M	2	Early LTx
2^a^	—	F	9	Slow course; no Tx
3^a^	—	M	2	Slow course; no Tx
4	—	M	Birth	Slow course; no Tx
5^b^	c.575G>A (hom.)	M	3	Late LTx
6^b^	c.575G>A (hom.)	M	Birth	Late LTx
7	c.638‐2delA (hom.)	M	36	Slow course; no Tx
8^c^	c.926_927del (hom.)	F	3	Early LTx
9^c^	c.926_927del (hom.)	M	Birth	Severe course with death
10^d^	Exon 1 deletion (hom.)	M	Birth	Slow course; no Tx
11^d^	Exon 1 deletion (hom.)	F	Birth	Slow course; no Tx
12	c.440 G>A, c.278_280del	F	2	Slow course; no Tx
13^e^	—	M	Birth	Slow course; discussed KTx
14^e^	c512_514delCCT (hom.)	M	Birth	Slow course; discussed KTx
15	c574c>T (hom.)	M	2	Slow course; no Tx
16	—	M	Birth	Severe course with death

Abbreviations: hom.: homozygous; LTx: liver transplant; KTx: kidney transplant.

^a^ sibling pair.

^b^ sibling pair.

^c^ sibling pair.

^d^ sibling pair.

^e^ sibling pair.

Variant pathogenicity was assessed in collaboration with experienced clinical geneticists and interpreted according to ACMG/AMP recommendations, taking into account the known loss‐of‐function disease mechanism of *TALDO1*. The variants c.512_514delCCT (homozygous) and c.575G>A (homozygous) had been previously reported as pathogenic in patients affected by transaldolase deficiency. In addition, for the c.512_514delCCT (homozygous) variant, functional validation was performed, showing a residual transaldolase enzymatic activity of < 1% in cultured fibroblasts, supporting its pathogenic role. Other variants identified in our cohort mainly consisted of truncating variants, canonical splice‐site variants, or exon deletions, all consistent with a loss‐of‐function effect. Their pathogenicity was defined based on a combination of criteria, including predicted functional impact, localization in conserved or functionally relevant regions, absence or extreme rarity in population databases, previous reports in the literature, homozygosity in the context of a highly specific clinical phenotype, and segregation data when available.

For patients in whom a complete genetic report was unavailable, the diagnosis of transaldolase deficiency was confirmed biochemically, particularly through the detection of a characteristic polyol profile on urinary polyol analysis, consistent with transaldolase deficiency.

The geographic origins of our patients were different: France, Poland, Türkiye, Cambodia, the United Arab Emirates, another country in South Asia, and several African countries. In one patient followed since childhood for “cryptogenic cirrhosis,” the molecular diagnosis was only confirmed in adulthood.

### 3.1 Liver

Liver involvement was present in all 16 patients (Table 3), with a median age at symptom onset of 1.3 months and presentation at birth in eight patients. Micronodular cirrhosis developed in 10 patients. Nearly all (*n* = 15) developed clinical portal hypertension (PH), initially splenomegaly, followed by esophageal varices in five patients and ascites in four. Coagulation disorders were observed in almost all patients, with a median age at detection of 1.3 months (six from birth). Hepatomegaly was universal, and only one patient lacked splenomegaly. No patient developed HCC, although one had a suspicious nodule as indication for Tx.

**TABLE 3:** Liver involvement.

Patient (No.)	Age at liver involvement (months)	Cirrhosis/fibrosis degree	Age at jaundice	Age at clinical portal hypertension (months)	Age at ascites (years)	Age at PT < 70% (months)
1	2	Cirrhosis	—	5	0.8	5
2	9	Cirrhosis	—	9	—	9
3	2	Cirrhosis	—	2	—	—
4	Birth	Cirrhosis	—	Birth	—	2
5	3	Cirrhosis	Birth	3	4	60
6	Birth	Cirrhosis	Birth	Birth	5	24
7	36	Cirrhosis	—	36	—	—
8	3	(Early death)	5 months	5	0.4	3
9	Birth	(Early death)	—	Birth	—	Birth
10	Birth	Cirrhosis	Birth	Birth	—	Birth
11	Birth	Cirrhosis	Birth	Birth	—	Birth
12	2	F0	—	2	—	—
13	Birth	F1	Birth	—	—	Birth
14	Birth	Cirrhosis	Birth	Birth	—	Birth
15	2	F1	Birth	48	—	—
16	Birth	(Early death)	Birth	Birth	—	Birth
*N* (%)	16 (100)	12 (75)	9 (56)	15 (94)	4 (25)	12 (75)
Median age (IQR)	1.3 (0.5–2.3)			2 (0.5–5)	2.4 years (0.7–4.3)	0.5 (0.5–7)

Abbreviations: —: not present; F0: absent fibrosis; F1: mild fibrosis; PT: prothrombin time.

### 3.2 Kidney

Kidney involvement was frequent (81%) but appeared later, with a median age at diagnosis of 4 years (Table 4). Two patients had neonatal kidney failure; one had congenital right kidney hypoplasia and left kidney dysplasia, developed severe vesicoureteral reflux, and underwent bilateral Cohen ureteral reimplantation. Tubulopathy was a common finding (68%), and five patients received ACE inhibitors or angiotensin receptor blockers for proteinuria. Five patients developed chronic kidney disease (CKD). In the two oldest ones (Patients 13 and 14), a progressive deterioration was observed; for these two patients, kidney transplantation was discussed. However, given the subsequent stability of glomerular filtration rate and biochemical parameters during FU, transplantation was not pursued, as criteria for end‐stage renal disease were not fulfilled.

**TABLE 4:** Kidney involvement.

Patient (No)	Kidney abnormality	Age at discovery (years)	CKD (GFR)	Tubulopathy with proteinuria	ACE‐inhibitors/sartans treatment
1	−	−	−	−	−
2	+	12	−	+	+
3	+	14	−	+	+
4	−	−	−	−	−
5	+	4	−	+	−
6	+	5	−	+	−
7	+	12	GFR 49	+	+
8	+ (PKD)	0.2	− (Early death)	−	−
9	− (Early death)	−	−	−	−
10	+	14	GFR 65	+	+
11	+	12	GFR 60	+	−
12	+	0.5	−	+	+
13	+ (CoKD)	Birth	GFR 30	−	−
14	+	Birth	GFR 41	+	−
15	+	2	−	+	−
16	+	Birth	− (Early death)	+	−
*N* (%)	13 (81)		5 (31)	11 (69)	5 (31)
Median age (IQR)		4 years (0.2–12)			

Abbreviations: +, present; −, not present/altered; CKD, chronic kidney disease; CoKD, congenital kidney dysplasia; GFR, glomerular filtration rate (mL/min/1.73 m^2^); PKD, polycystic kidney disease.

### 3.3 Hematologic Involvement

All patients exhibited cytopenia at clinical onset (two with isolated thrombocytopenia and the remainder with bi‐ or tri‐cytopenia). Twelve patients (75%) developed chronic cytopenia (one with isolated thrombocytopenia and the others with bi‐ or tri‐cytopenia). Thrombocytopenia was the most frequent finding, although splenomegaly was observed in 15 patients. Independently of thrombocytopenia, four patients had bleeding tendencies: two siblings experienced major intracranial hemorrhages (a subgaleal hematoma complicated by a compressive intraorbital hematoma, and an extradural hematoma following head trauma). Four patients also developed transient hemolytic anemia.

### 3.4 Heart

Cardiac abnormalities were present in most patients (88%). The most common one was a persistent patent foramen ovale (PFO), which was surgically closed in three patients. Other findings included ventricular septal defects (VSDs) in two patients and aortic root anomaly and patent ductus arteriosus in two others.

True cardiomyopathy was diagnosed in five patients: two hypertrophic cases detected prenatally, two diagnosed at birth (one dilated and one hypertrophic), and one dilated cardiomyopathy diagnosed in the first year of life. One patient only (with postnatal dilated cardiomyopathy) developed chronic heart failure with an ejection fraction of 40% requiring therapy. Another patient with neonatal dilated cardiomyopathy improved within the first 4 months of life with diuretics and digoxin. No medical treatment was required in the remaining cases. One patient with prenatal cardiomegaly developed pulmonary hypertension with a patent ductus arteriosus, which resolved spontaneously after 1 month.

### 3.5 Endocrine Involvement

Hypergonadotropic hypogonadism was the most frequent endocrine finding (*n* = 7; six males), diagnosed either in the first year of life (*n* = 5) or at puberty (*n* = 2). In one patient with a severe disease course who died early, hypogonadism was identified at birth. Cryptorchidism was seen in four patients and micropenis in two. Five male patients received androgen therapy, and one female patient received estrogen–progestin therapy. Additionally, three patients had a growth delay, and a sibling pair developed secondary adrenal cortisol insufficiency and primary hypothyroidism requiring replacement therapy.

### 3.6 Neurological Involvement

Eight patients (50%) showed normal cognitive development, while the others presented mild delay. Overall, patients achieved good school and work integration, with autonomy comparable to that of the general population; brain MRI was not systematically performed in all cases. In one sibling pair, peripheral axonal neuropathy was diagnosed, with absent patellar reflexes confirmed by electrophysiologic studies. Neurosensory hearing loss was documented in one patient.

### 3.7 Pulmonary Manifestations

One patient had chronic respiratory problems, including oxygen requirement during infections from the first months of life and after liver transplantation (LTx); no relationship was found between liver involvement and leukocyte counts.

### 3.8 Overall Clinical Course Over Time

During FU, hepatic involvement most frequently manifested early in life. In several patients, liver disease initially presented with liver failure, while in others early hepatic dysfunction partially improved before progressing toward cirrhosis. In the majority of patients, despite the development of cirrhotic changes, liver function remained stable over time and did not progress to an indication for transplantation. Renal involvement was characterized mainly by tubulopathy with proteinuria, which remained stable under medical treatment in most patients. In contrast, a progressive decline in renal function was observed in a subset of individuals during FU. Hematologic abnormalities were present throughout the disease course, with cytopenias persisting over time; thrombocytopenia was the most consistently observed abnormality. Cardiac involvement most commonly consisted of PFO, which required closure in some cases. Structural or functional myocardial abnormalities were observed predominantly during the prenatal period or early infancy and were rarely detected later in life. Endocrine involvement developed over time in several patients, with hypergonadotropic hypogonadism remaining stable under hormone replacement therapy. Additional endocrine deficiencies, including secondary adrenal insufficiency and primary hypothyroidism requiring replacement therapy, were observed only in a sibling pair. Regarding neurological involvement, long‐term survivors most frequently showed mild intellectual delay during FU, without progression to severe neurocognitive impairment.

### 3.9 LTx Outcomes

Growth failure posed a major challenge in the three most severely affected patients; parenteral nutrition offered minimal improvement, and weight remained less than 4 kg. Two children died before Tx became possible, and the third one died of Tx‐related complications (Patient 8). Overall, four patients underwent LTx (median age 7 years; range 0.5–15 years) with a median posttransplant FU of 5.3 years. Indications included: early severe liver failure in two neonates (Patients 1 and 8), progressive liver dysfunction in a 12‐year‐old patient (Patient 5), and suspected HCC in a 15‐year‐old patient (Patient 6, not confirmed on pathology).

Patient 5 remains currently in good general condition, liver function is preserved and renal function is normal. No cytopenia is currently observed. At the age of 17, the patient developed headaches, dizziness, and transient visual disturbances, followed later by persistent diplopia without ocular pain or ophthalmoplegia. Brain MRI and CT showed no focal lesions, with only mild chronic ventricular enlargement and slightly widened cortical sulci. Ophthalmological examination revealed strabismus, while earlier fundoscopy was essentially normal. At present, dizziness and visual darkening have resolved, headaches occur only occasionally and respond to analgesics, and diplopia persists intermittently. Patient 6 actually remains with multiple chronic comorbidities including incisional hernia, short stature, delayed puberty, and renal tubulopathy. However, his liver graft function is stable, with histology at the borderline of normal and no features of acute cellular or humoral rejection or fibrosis. Renal function is impaired, with elevated creatinine and cystatin C levels and evidence of progressive renal dysfunction. He exhibits significant proteinuria despite the absence of arterial hypertension on ambulatory blood pressure monitoring. Acid–base disturbances have been noted, consistent with underlying renal tubular dysfunction. Renal Doppler ultrasound shows no renal artery stenosis but reduced vascularization of the renal parenchyma. Overall, he is in good general condition but requires ongoing nephrological evaluation due to gradually worsening kidney function. The third patient who underwent LTx (Patient 1) developed early EBV‐driven polymorphic posttransplant lymphoproliferative disease and was treated with four rounds of rituximab, followed by repeated doses of intravenous immunoglobulin. At latest FU, the patient had normal kidney function, as measured by iohexol clearance and cystatin C, normal levels of hemoglobin, platelets and white blood cells, and normal thyroid levels. Cortisol levels were very low, consistent with secondary adrenal insufficiency due to prolonged treatment with prednisolone. However, liver function remains preserved.

LTx was not indicated for the remaining patients.

## 4 Discussion

We described the multiorgan involvement in a European cohort of patients with TALDO deficiency, with long‐term FU exceeding 20 years.

In our cohort, liver involvement was predominant and present in all patients at diagnosis, followed by hematologic, cardiac, endocrine, and renal involvement. Our experience showed that, in patients with severe multiorgan involvement during the first months of life, severe growth failure unresponsive to parenteral nutrition could be the main reason LTx was not performed. This aspect may be linked to molecular features of cell growth still unknown in TALDO deficiency.

A selection bias should nevertheless be considered, as all patients were diagnosed at pediatric hepatology centers; milder forms of the disorder without hepatic involvement probably exist.

The multiorgan involvement of TALDO deficiency reflects the crucial role of the PPP in cellular proliferation and growth through the production of R5P, which is required for DNA and RNA synthesis. In addition, the PPP is essential for generating NADPH, and its depletion leads to secondary glutathione depletion and increased oxidative stress. It is therefore not surprising that the liver (detoxification and synthesis) and bone marrow (hematopoiesis) are among the most affected organs [3, 4].

As observed in our patients, TALDO deficiency can present antenatally, often with intrauterine growth restriction, classifying it as an embryo‐fetopathy. A case of meconium ileus with prenatal ultrasound signs, postnatal surgical resection, and ileostomy has been reported [5], underscoring the importance of the PPP in prenatal life. However, milder phenotypes of this disease have also been described [6, 7].

Our study focused on the course of hepatic involvement, a consistent feature of TALDO deficiency. Based on our data, we identified different phenotypes: a severe form leading to death before transplantation, a rapidly progressive form requiring early transplantation, a slower course where transplantation is considered before age 20, and a group with a slow course and no organ failure in whom transplantation is not necessary.

In patients with maintained organ function, cirrhotic evolution was frequent. None of our patients developed HCC, although this has been described in the literature, possibly linked to the enzyme's role in the mTOR pathway [8].

In our cohort, four patients underwent LTx for different indications; one patient died due to postoperative complications, while three are alive at last FU. The detailed clinical course of two of the surviving patients has previously been reported by Stefanowicz et al. [9]. Since the publication of this study, posttransplant outcomes in the two patients have remained overall stable, with preserved hepatic function. The neurological–ophthalmological symptoms developed by the first patient showed an uncertain relationship to both the underlying disease and the transplantation. In the second patient, the worsening of renal function may be partially attributable to immunosuppressive therapy, given its known nephrotoxic effects. In the third living patient, the transplant outcome has been optimal despite the development of a hematological complication. Overall, our data from the three surviving transplanted patients suggest that LTx is effective in transaldolase deficiency, although its benefits appear to be primarily limited to hepatic function, whereas the impact on preexisting extrahepatic complications, particularly renal and endocrine manifestations, seems more limited. Future studies including larger cohorts of transplanted patients will be necessary to determine whether earlier transplantation may influence the development of extrahepatic complications. With regard to cytopenias, in our experience these tended to improve after transplantation, likely secondary to improved hepatic function.

Data on LTx for this disease remains limited; six patients worldwide have been reported to have undergone transplantation for this disease [7, 10, 11, 12, 13]. However, according to our experience, LTx remains a life‐saving option, and if indicated it should possibly be performed early.

In patients with a milder disease, growth curves were generally normal, with only isolated cases of mild and transient growth delay.

Compared with Williams et al. (four patients overlapped with this cohort), the longer FU in our cohort revealed greater renal involvement, with several patients developing CKD and progressive organ failure in addition to tubulopathy with proteinuria, the most commonly reported renal finding in this disease [4]. Progressive kidney dysfunction, which appeared later than liver disease, can be severe and may lead to end‐stage renal disease requiring kidney transplantation. Kidney transplantation has not yet been reported in patients with TALDO deficiency. These findings underscore the importance of periodic monitoring of glomerular filtration in affected individuals.

Hypergonadotropic hypogonadism is a feature of the disease, with a high prevalence in our cohort [2]. Williams et al. also described patients with thyroid insufficiency and growth hormone deficiency [4]. In our cohort, secondary adrenal insufficiency was identified in a sibling pair, whereas no hypothalamic–pituitary–adrenal axis abnormalities were reported in the Williams cohort. This new finding suggests that decreased NADPH in TALDO deficiency may also impair hormone production at the hypothalamic or pituitary level, not solely at the gonadal level. This should therefore be carefully monitored during FU.

Our patients' cardiac involvements are consistent with Williams et al. [4] and Eyaid et al. [1]. Persistent PFO predominated among cardiac abnormalities, but VSDs and various forms of cardiomyopathy were also observed, sometimes evident prenatally. However, one patient only required prolonged treatment for myocardial dysfunction, suggesting a limited impact on the prognosis of this disease. The high prevalence of cardiac involvement is likely related to the role of transaldolase in nucleic acid synthesis [3, 4].

Hematologic manifestations, including coagulopathy and cytopenias (mainly thrombocytopenia but also pancytopenia), were the most frequent findings during long‐term FU. Almost all patients had hypersplenism, likely influencing cytopenia. It is therefore plausible that transient bone marrow insufficiency is a major determinant of cytopenia in the early stages of the disease, but less so later on. Chronic neutropenia or leukopenia was not associated with an increased risk of severe infections.

Coagulopathy, a sensitive indicator of liver disease progression, was linked to severe hemorrhagic episodes (including intracranial hemorrhages) after trauma; extra caution is required for surgical interventions in these patients [14].

None of our patients developed severe cognitive impairment, and only mild intellectual delay was observed. Cognitive outcomes in TALDO deficiency remain poorly described in the literature.

One patient only developed chronic lung disease, requiring frequent oxygen supplementation during infections. Few cases of pneumopathy, potentially linked to hepatopulmonary syndrome, have been reported [1, 15].

Regarding genotype–phenotype correlation, despite the limited number of patients, some observations can be made. Such a correlation does not appear straightforward for the c.575G>A (homozygous) variant, particularly with respect to liver disease progression; in fact, this variant was shared by Patients 1, 5, and 6, all of whom underwent LTx for different indications. Limited to the single young patient currently available, the combination of c.440G>A and c.278_280del appeared to be associated with a mild hepatic phenotype, without cirrhosis or liver failure. A similar phenotype was observed in the patient carrying the c.574C>T (homozygous) variant. In contrast, the sibling pair carrying the Exon 1 deletion (homozygous) presented a highly similar disease phenotype, characterized by early‐onset cirrhosis, severe hemorrhagic manifestations, secondary adrenal cortisol insufficiency, and primary hypothyroidism, not observed in other patients.

The sibling pair with the c.926_927del (homozygous) variant exhibited a very severe and early‐onset disease course, leading to death in one case and to LTx in the other, followed by death due to posttransplant complications. Finally, in the sibling pair carrying the c.512_514delCCT variant, a similar renal phenotype was observed, whereas hepatic involvement differed, with mild fibrosis in Patient 13 and overt cirrhosis in Patient 14. Overall, although additional data are needed, some variants appear to show a genotype–phenotype correlation, particularly when considering extrahepatic complications observed in certain sibling pairs.

## 5 Conclusion

We describe the long‐term outcomes of multiorgan complications in TALDO deficiency. Although the disease phenotype is well known, there is limited information regarding its long‐term evolution. This is the longest FU report of a multicenter cohort of patients with this disorder.

Based on liver involvement, disease progression can be categorized into distinct severity phenotypes. Hepatic, hematologic, and renal complications appear to be the most relevant for patient prognosis, whereas cardiac involvement seems to have less prognostic impact. Neurocognitive outcomes were generally favorable.

Although the limited cohort size precludes definitive genotype–phenotype correlations, our data suggest that certain variants may be associated with recurrent phenotypic patterns within families, while others show marked intragenotypic variability, particularly regarding liver disease progression.

LTx remains a life‐saving therapy for this disease and should be considered early when indicated for liver failure or HCC, as no other etiologic treatments are currently available. In the most severely affected young patients, inadequate weight gain may represent a major challenge for transplantation, as inadequate nutritional status may be a contraindication. The role of kidney transplantation in advanced CKD remains to be defined.

## Authors' Contribution

M.S. has been involved in data recording and writes the first and the revised version of the manuscript. A.B., A.W., F.F., C.D., T.L., M.G., U.C., I.J., J.P., P.S., T.C., B.F., D.S.B., and L.H.B. have carried out periodical clinical evaluations of the patients, managing the multidisciplinary approach, and contributing to data recording. M.S., M. Schiff, P.D.L., M.C.S., and F.L. have been involved in the manuscript review process; F.L., M. Schiff, and P.D.L. validated the final version of the manuscript.

## Funding

The authors have nothing to report.

## Ethics Statement

Our study adhered to the Declaration of Helsinki and was approved by the Institutional Review Boards at Necker University Hospital.

## Consent

For all the patients, a consent was signed at the same time of hospitalization, in addition to the permission of care. Written informed consent was obtained from the parents of each subject prior to genetic testing. Data from the Swedish patients were collected in line with ethical permit allowing us to extract chart data.

## Conflicts of Interest

The authors declare no conflicts of interest.

## Data Availability

The data that support the findings of this study are available on request from the corresponding author. The data are not publicly available due to privacy or ethical restrictions.
